# How Are the Interests of Incapacitated Research Participants Protected through Legislation? An Italian Study on Legal Agency for Dementia Patients

**DOI:** 10.1371/journal.pone.0011150

**Published:** 2010-06-16

**Authors:** Sabina Gainotti, Susanna Fusari Imperatori, Stefania Spila-Alegiani, Laura Maggiore, Francesca Galeotti, Nicola Vanacore, Carlo Petrini, Roberto Raschetti, Claudio Mariani, Francesca Clerici

**Affiliations:** 1 National Centre for Epidemiology, Surveillance and Health Promotion, National Institute of Health, Rome, Italy; 2 Chair of Neurology, Centre for Research and Treatment on Cognitive Dysfunctions, University of Milan, “L. Sacco” Hospital, Milan, Italy; 3 Bioethics Unit, Office of the President, National Institute of Health, Rome, Italy; The Kenya Medical Research Institute, Kenya

## Abstract

**Background:**

Patients with dementia may have limited capacity to give informed consent to participate in clinical research. One possible way to safeguard the patients' interests in research is the involvement of a proxy in the recruitment process. In Italy, the system of proxy is determined by the courts. In this study we evaluate the timing for appointment of a legal proxy in Italy and identify predictive variables of appointment.

**Methodology/Principal Findings:**

Subjects were recruited among the outpatients seeking medical advice for cognitive complaints at the Centre for Research and Treatment of Cognitive Dysfunctions, University of Milan, “Luigi Sacco” Hospital.

The Centre was participating to the AdCare Study, a no-profit randomised clinical trial coordinated by the Italian National Institute of Health. The requirement that informed consent be given by a legal representative dramatically slowed down the recruitment process in AdCare, which was prematurely interrupted. The Centre for Research and Treatment of Cognitive Dysfunctions collected data on the timing required to appoint the legal representatives. Patients diagnosed with dementia and their caregivers were provided information on the Italian law on legal agency (law 6/2004). At each scheduled check-up the caregiver was asked whether she/he had applied to appoint a legal proxy for the patient and the time interval between the presentation of the law, the registration of the application at the law court chancellery and the sentence of appointment was registered. The study involved 169 demented patients. Seventy-eight patients (46.2%) applied to appoint a legal proxy. These subjects were usually younger, had been suffering from dementia for a longer time, had less than two children and made more use of memantine. The mean interval time between the presentation of the law and the patients' application to the law court chancellery was two months. The mean interval time between the patient's application to the law court chancellery and the sentence of appointment was four months.

**Conclusions/Significance:**

In Italy the requirement that legal representatives be appointed by the courts slows down subjects' participation in research. Other procedures for legal agency of the incapacitated patients may be adopted, taking as examples other EU countries' systems.

## Introduction

People with dementia often lack mental capacity and subsequently need assistance in their decision making. Research involving individuals with compromised mental ability can be ethically challenging as the lack of capacity may limit their ability to give free and informed consent.

The need to adopt special cautions in research involving individuals with compromised capacity has been highlighted by the most relevant declarations on research ethics, like the Nuremberg Code and the Declaration of Helsinki.

The Nuremberg Code of 1947 [1], adopted a restrictive approach towards participation of incompetent patients in research, stating that: “The voluntary consent of the human subject is absolutely essential. This means that the person involved should have legal capacity to give consent” (point 1).

In the 1964 version of the Declaration of Helsinki [2] the possibility of “surrogate” or “proxy” consent overcame the “preclusion” from participation in research of incompetent patients: “In case of legal incompetence, informed consent should be obtained from the legal guardian in accordance with national legislation” (point 11).

Thereafter, following the first version of the Declaration of Helsinki, other relevant declarations on research ethics have confirmed the acceptability of surrogate or proxy consent thus sanctioning the ethical acceptability of participation of incompetent adults in research, provided that more protections be offered to these subjects.

The 2008 version of the Declaration of Helsinki states that potential research subjects who are incompetent “must not be included in a research study that has no likelihood of benefit for them unless it is intended to promote the health of the population represented by the potential subject, the research cannot instead be performed with competent persons, and the research entails only minimal risk and minimal burden” (point 27).

Moreover, “When a potential research subject who is deemed incompetent is able to give assent to decisions about participation in research, the physician must seek that assent in addition to the consent of the legally authorized representative. The potential subject's dissent should be respected” (point 28).

These principles have been adopted by all national legislation in western countries.

However, while the protection afforded to potential research participants who lack capacity has received recognition through legislation and ethical debate, the practical aspect of recruiting and retaining such participants in research presents a number of challenges.

One main problem in dementia research is the evaluation of the patient's capacity and, when the individual is deemed competent, the requirement that he or she expresses a valid consent to participate. To give valid consent the potential participant should understand and retain relevant information, weigh the information, make a decision and communicate the decision [3]. In the context of clinical trials the information should include the purpose of the trial, the trial procedures, the risks and benefits of participation. Potential participants should understand the concept of equipoise which provides the ethical basis for the conduction of a clinical trial, placebo (if used), and randomization.

According to some authors if participants were required to understand all of this information, dementia research would become “harder to conduct” [4] or it would cease entirely, restricting the continuing development of a much needed area of research [5].

As indicated by the Declaration of Helsinki, one possible way to meet the ethical requirements of informed consent is to safeguard the potential participants interests through the use of proxy consent in the recruitment process. At present, the majority of Alzheimer's Disease (AD) research is conducted with “double consent”, that is, by obtaining consent from both the patient and a proxy who is typically a family caregiver.

The practice of obtaining surrogate consent however can vary according to differences in national legislation. In particular, in some countries, including Italy, the system of proxy is determined by the courts - a procedure which is not necessarily required for the recognition of a proxy in other countries.

In the European Union (EU) a common legal framework for the inclusion and protection in research of adults who lack capacity is set up by the Directive 2001/20/EC [6], also known as the “Clinical Trials Directive” (hereinafter the Directive).

To ensure legal protection to incapacitated participants in research the Directive requires the written consent of the participant's legal representative.

However, according to the Directive “The notion of legal representative refers back to existing national law and consequently may include natural or legal persons, an authority and/or a body provided for by national law”(introduction, point 5).

National implementation of the Directive hence raises distinct issues which reflect the legal, cultural, political and socio-economic background within each member state.

To compare the national legal framework of some EU member states regarding the inclusion and protection in research of incapacitated participants we retrieved national laws using the European Forum for Good Clinical Practice (EFGCP) Report on “The Procedure for the Ethical Review of Protocols for Clinical Research Projects in the European Union” [7].

In particular, we analysed the national laws on clinical trials which are written in English, French or Spanish or for which an English translation is available.

Some countries have legislation that is specific to an incapacitated participant's involvement in research while in other countries the proxy provision falls from legislation on health and welfare more broadly.

Several legislation provide that an individual is able to appoint a representative prior to the onset of incapacity (e.g. Belgium, France, Italy, U.K. and Germany).

In all of the analysed legislation: Belgium [8], Denmark [9], Finland [10], France [11], Germany [12], Ireland [13], [14], Italy [15], [16], the Netherlands [17], Spain [18] and the UK [19], [20] a relative is allowed to take on the role of proxy.

However, only in Germany and Italy the system of proxy is determined by the courts - a procedure which is not necessarily required for the recognition of a proxy in other member states.

In Belgium, the Netherlands, Finland and Spain a more pragmatic procedure has been adopted for proxy consent in research. This system describes a cascade of measures aimed to legitimately arrive at a consent that reflects the individual's presumed will, going from the authorised legal representative to a hierarchy of family members.

In France a similar system for legal agency is in place. However, if according to the ethics committee the research imposes serious risks to the participant's private life or bodily integrity, authorisation to participation must be given by the tutelary judge.

In Ireland an incapacitated person may only participate in a clinical trial if written and signed consent is given by a person, independent from the trial, who in the opinion of the ethics committee is able to give a decision on such a participation.

In Denmark, if an adult is permanently incapacitated consent must be given by the nearest relative (or legal representative) and the person's general practitioner (GP).

Also in the UK, if it is not reasonably practicable to contact an adult's legal representative then the doctor primarily responsible for the medical treatment of the individual, or a person nominated by the relevant health care provider, can act as legal representative, providing they have no connection with the conduct of the clinical trial [19].

The different ways of obtaining surrogate consent for subject's participation in research in the EU countries may have an impact on countries “attractiveness” for dementia research.

**Figure 1:**
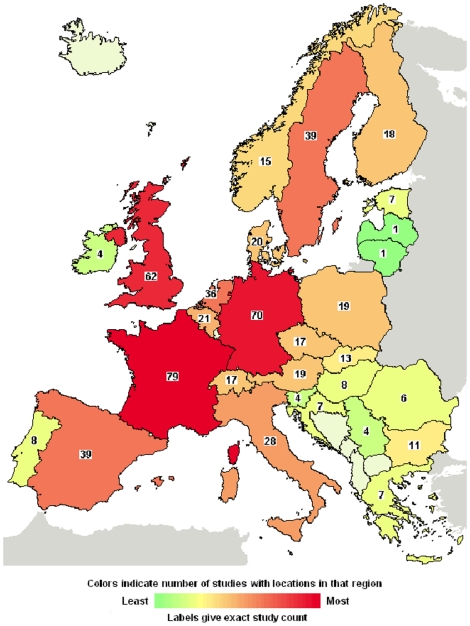
Interventional Studies on dementia in Europe (Available: www.clinicaltrials.gov; accessed 2010 May 3).

For example, one may suppose that countries where courts are not involved in the appointment of a patient's proxy are more attractive for dementia research, as courts involvement may slow down the process of appointment of the proxy and hence of obtainment of the informed consent. However the available data, even if they are quite limited, do not confirm this hypothesis. According to clinicaltrials.gov many clinical trials on dementia are being conducted in Europe (Figure 1).

It is to note that in Germany, where the patient's legal proxy must be appointed by the courts, the number of clinical trials involving patients with dementia is high.

Recently in Germany a law was published, amending the Civil Code, regulating sensitive issues like the value of advanced directives and surrogate consent in the care of incompetent patients. The law specifies the value and the limits of the decisions of the “authorised representatives” in the care of the incompetent patient, but does not describe the process of appointment of the authorised representative [21].

Unluckily we did not retrieve detailed information on the German system for legal agency of persons with compromised capacity, which apparently allows a good level of participation in research of the patients with dementia.

This is not the case for Italy.

In Italy relatives are not “legally authorized representatives” and cannot give informed consent in clinical practice nor in medical research. Legal agency is mediated by the courts but at present very few patients have appointed a legal representative. This may be due to the fact that, until recently, legal agency for incompetent persons was synonymous of debarment.

In 2004 a law has changed the civil code, and in place of the traditional institutions of guardianship and tutelage, which completely deprived persons of their legal rights, legal agency is now focused on care and assistance [16]. The new legal entity is a “carer” (in Italian: “amministratore di sostegno”) and is appointed by the tutelary judge, found in every magistrate's court. The law provides for a hierarchy of family members who can be appointed as legal proxy going from the beneficiary's spouse to his or her partner, father or mother, son or daughter, brother or sister, and other persons who are close to the patient. Nomination is inexpensive, the patient can directly apply for it and he/she can indicate his or her legal proxy prior to the onset of incapacity. Also relatives, neighbours and healthcare professionals can apply for appointment. Persons working in the healthcare or social services who are directly involved in the care of the person, when needed, must invite the tutelary judge to appoint a legal proxy for the patient. However, they themselves cannot be appointed if they are involved directly in the care of the patient.

The legal proxy must warrant the patient's desires in treatment decisions.

In the act of appointment, the judge must specify all actions that the legal proxy will be able to do for the person, which may include the care of his or her health and the right to give informed consent, as well as the management of his or her financial affairs.

The kind and number of powers and duties assigned to the legal proxy do not determine the length of the process of appointment which, according to the law, should be completed in 60 days.

However, in very urgent cases the tutelary judge may intervene with an accelerated procedure.

Theoretically, the law is easily applicable. The patient or his or her relatives do not need a lawyer's assistance to enforce it, they do not have to pay any money (apart from a € 8 revenue stamp) but must present several documents to the tutelary judge among which a medical certificate and a birth certificate which can only be obtained in the patient's common of birth.

Since the publication of the law 6/2004 many Italian courts have experienced an exponential increase in demand of appointment of the legal proxies for the persons who need it [22].

For this reason we hypothesise that the requirement that informed consent for an incapacitated subject's participation to a research be given by a legal representative appointed by the courts slows down the recruitment process in research thus complicating the conduction of dementia research in Italy.

In this study we want to verify how does the Italian law 6/2004 work and more precisely:

1. Evaluate the timing and procedure for the appointment of a patient's legal proxy according to the law 6/2004;
2. Identify the predictive variables of the appointment of the legal proxy according to the law 6/2004.

## Methods

Subjects were recruited among the outpatients seeking medical advice for cognitive complaints at the Centre for Research and Treatment of Cognitive Dysfunctions of the Department of Neurology, University of Milan, “Luigi Sacco” Hospital.

The centre was participating to the AdCare Study, a no-profit randomised clinical trial coordinated by the Italian National Institute of Health on the efficacy and safety of antipsychotic drugs in the treatment of behavioural and psychological disturbances in patients with Alzheimer's disease (AD) (the AdCare Study, Eudract code: 2008-000243-33).

The AdCare study was approved by the ethics committee of the Italian National Institute of Health and by the local research ethics committees (RECs) of the 19 clinical centres which participated in the study, including Luigi Sacco Hospital's REC.

The procedure to obtain informed consent in AdCare was quite elaborated. First, subject's competence was evaluated by means of the Mini Mental State Examination (MMSE). If the subject's score was ≥20, then he or she underwent four additional neuropsychological tests: the Babcock Recall Story Test, the Controlled Oral Word Association (letters and categories) and the Trail making test. If the subject's score to the four tests was higher than the established cut-offs the subject was deemed able to give informed consent and was informed of the study characteristics: its purposes, design, possible risks and benefits of participation, voluntariness of participation and the right to withdraw [23].

After presenting the study some checking questions were made to the participant to assess his or her level of comprehension of the information received [3]. The questions were:

What do you believe is wrong with your health now?

Do you believe that you need some kind of treatment?

What is treatment likely to do for you?

What makes you believe it will have that effect?

What do you believe will happen if you are not treated?

Why do you think your doctor has (or I have) recommended this treatment?

Have you decided whether to follow your doctor's (or my) recommendation for treatment? Can you tell me what that decision is?

(if no decision) What is making it hard for you to decide?

If the subject's MMSE score was <20, adjusted for age and education, or if the subject's score to the other four tests was lower than the established cut-offs, the subject was deemed unable to give informed consent and consent had to be given by a legally authorised representative.

The requirement that informed consent be given by a legally authorised representative dramatically slowed down the recruitment process in AdCare. The Centre for Research and Treatment of Cognitive Dysfunctions at the Milano Sacco Hospital decided to collect data on the timing required to appoint a legal representative for subjects-participants to document the real difficulty of the process.

Patients included in this survey were enrolled from September 2007 to October 2009. All patients underwent a diagnostic work-up, routinely applied in the outpatient clinic for evaluation of patients with cognitive impairment, which included an interview with the patient and an informant, medical, psychiatric and neurological examinations, routine blood screening, extensive neuropsychological examination and structural neuroimaging.

Two experienced neurologists (FC and LM) collected information concerning cognitive and behavioural symptoms (psychosis, agitation, sleep disorders) and assigned Basic [24] and Instrumental [25] Activities of Daily Living (BADL, IADL) and Clinical Dementia Rating (CDR) [26] scores. The information for the BADL, IADL and CDR was collected according to the subject and an informant. Global cognitive functioning was assessed using the MMSE [27].

Dementia was diagnosed according to DSM IV criteria [28], Alzheimer's Disease (AD) according to NINCDS-ADRDA criteria [29], Lewy Body Dementia (LBD) according to McKeith criteria [30], Frontotemporal dementia (FTD) according to Lund and Manchester criteria [31] and vascular dementia (VaD) according to NINDS-AIREN criteria [32].

Patients were all receiving standard treatments for dementia, including cholinesterase inhibitors (AchE-Is), memantine, antipsychotics, antidepressants and benzodiazepine.

All the consecutive patients receiving a diagnosis of dementia and their caregiver were included in the study and were asked whether they were acquainted with the law 6/2004, regarding the possibility of appointing a legal proxy for the patient. If they were not acquainted with the law 6/2004 a neurologist (FC or LM) and a psychologist (SFI) provided information on it.

The time dedicated to describe the law 6/2004 to each patient and family member was about one hour, to be added to the usual visit time.

The neurologist illustrated the clinical reasons why a person with dementia may present limitations of the decisional capacity, which may render necessary the appointment of a legal proxy. For example, language disturbs may affect the capacity to understand relevant information or to express one's wishes; memory disturbs may affect the capacity to retain the information to make an informed choice in clinical practice and in research.

Successively, the neurologist illustrated some clinical contexts in which the informed consent of a legal proxy would be required (e.g. invasive procedures and surgical acts, prescription of drugs for off label use, participation in clinical research). To end, the neurologist described the context of clinical research (the AdCare study) for which the informed consent of a legal proxy was required, underlining the importance of the study and its relevance for public health.

The accompanying family members were given time to read an informative sheet which resumed the law 6/2004 and the informed consent form of the AdCare study. The family members could keep a copy of the documents to discuss participation to the AdCare study with their general practitioner (GP) or with other family members.

Successively, during the same encounter, the psychologist described technical aspects of the law (for example the possibility that the tutelary judge extended the field of legal agency to the management of the patient's properties and to the duty to be accountable for the patient's expenses) and bureaucratic aspects (court's address, court's opening times, documents to be presented to the tutelary judge).

Moreover, the psychologist made herself available, also by giving her number of mobile phone, to answer to every question which may raise during the visit or thereafter, and to help the patient and the caregiver(s) in filling the court's forms. Every patient and caregiver was left the time to discuss these issues with their GP or with other persons whom they trusted.

Finally, the patient was provided with a disease certificate and was recommended to start up the procedure of appointment of the legal proxy specifying that the legal proxy must “keep contacts with the health authorities and give informed consent to surgical acts, medical treatments and participation in clinical research”.

During the following visits, the same neurologist and the same psychologist made themselves available to give other information and to listen to any patient's or caregiver's doubts.

At each scheduled check-up the caregiver(s) was/were asked whether he or she had applied to appoint a legal proxy and, if yes, progresses of the procedure were checked.

If the patient or the caregiver(s) had applied to appoint the legal proxy the following information were gathered:

1. date of the medical visit and presentation of the law;
2. date of registration of the application at the law court chancellery;
3. date of the sentence for the appointment of the legal proxy;
4. legal proxy's name and degree of relationship, if any, with the beneficiary;
5. legal proxy's powers;
6. legal proxy's duties.

If no legal proxy was appointed, we asked to the patient and the caregiver(s) why they decided not to apply for appointment.

This survey was a non interventional study, and the approval of a research ethics committee was not required by the Italian legislation. Hence, the approval of the local research ethics committee was not sought.

Nearly all the recruited patients were not able to give consent and the informed consent of a caregiver who is not a legally authorised representatives has no legal value in Italy. Therefore, informed consent was not required to subjects nor to their “non legally authorised” caregivers to participate in this survey. Indeed, for many patients the main “outcome” of the survey was the appointment of a legally authorised representative who will be able to give informed consent in future studies.

## Results

The study involved 172 demented patients, affected by AD (n = 133; 77%), VaD (n = 15; 9%) LBD (n = 12; 7%), FTD (n = 3; 2%) and unspecified dementia (n = 9; 5%), respectively.

**Figure 2:**
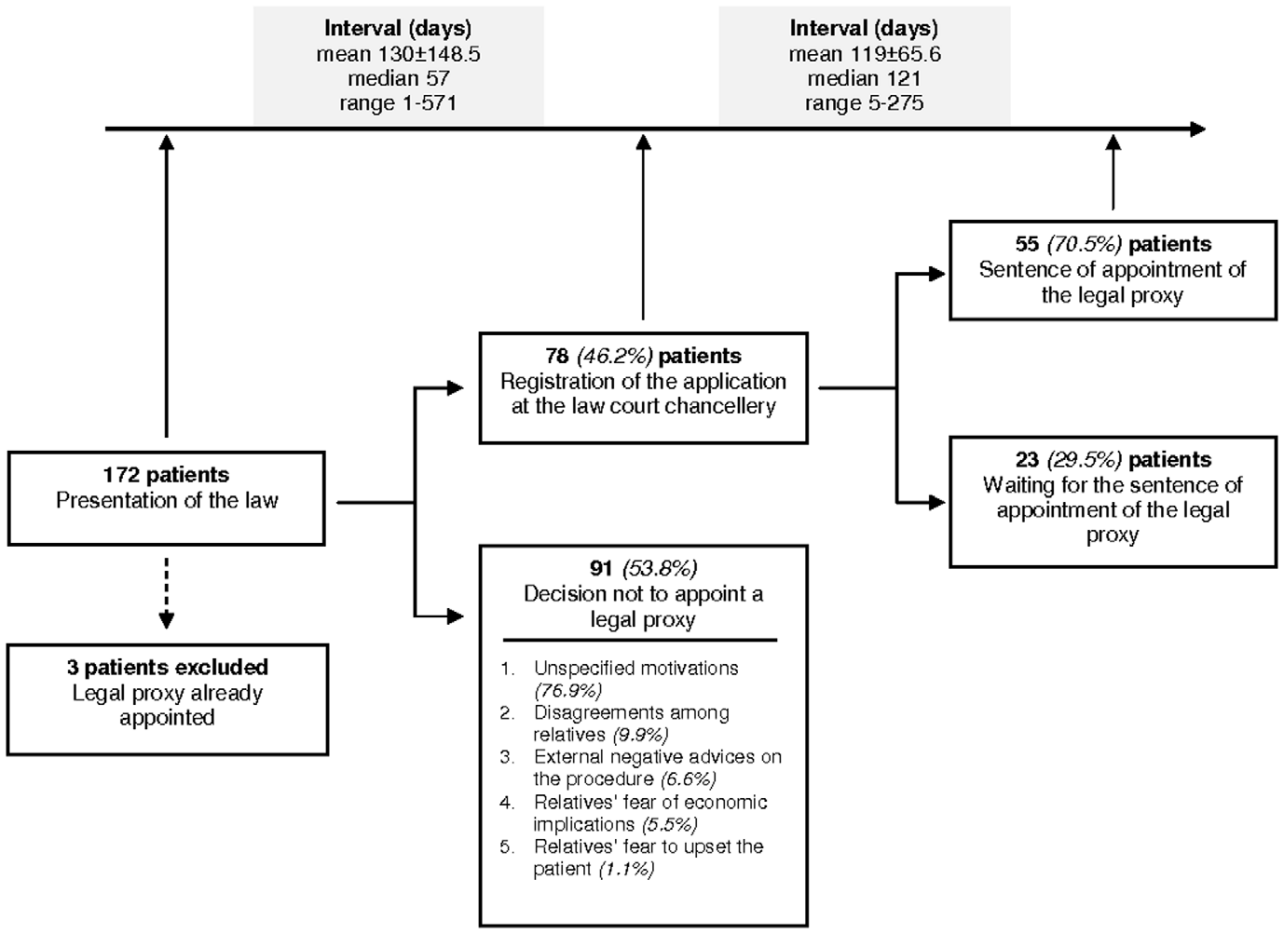
Patients applying and not applying to appoint a legal proxy and the time required for appointment.

Only 3 patients (1.7%) and their relatives were already acquainted with the law 6/2004 and had autonomously applied to appoint a legal proxy (Figure 2).

Seventy-eight patients out of 169 (46.2%) applied for appointment. Nearly two months passed between the date of the medical visit in which the neurologist and the psychologist presented the law to the patient and his or her caregiver(s) and the date in which the patient and the caregiver(s) applied to the law court chancellery (median time 57 days).

Fifty-five applications out of 78 (70.5%) ended up with appointment. An average time of four months passed between registration of the application to the law court chancellery and the sentence of appointment of the legal proxy (median time 121 days). In all these cases the tutelary judge appointed a relative: a son or a daughter, 63.6% (35/55); the spouse, 27.3% (15/55); others relatives (two nephews, one sister, one son-in-law, one sister-in-law), 9.1% (5/55).

Details on the cohort characteristics and description of the two subgroups of patients (those for whom a legal proxy was appointed and those for whom it was not appointed) are reported in figure 2.

Table 1 shows the demographic and clinical characteristics of the 169 patients included in the cohort and the two subgroups of patients. We found only two statistically significant differences between the two groups: the subjects who started up the procedure of appointment had been suffering from dementia for a longer time (2.3±2.2 versus 1.6±1.8 yrs; p = 0.02) and made more use of memantine (15.4% vs 4.4%; p = 0.02) than those who did not start up the procedure of appointment.

**Table 1:** Baseline patient characteristics.

	All patients n. 169	Application for the appointment of a legal proxy		p
		Yes (n. 78)	No (n. 91)	
Mean age (years)	79±7.2	79±7.7	80±6.7	0.18
Female (%)	66.3	67.9	64.8	0.67
Mean duration of disease (years)	1.9±2.0	2.3±2.2	1.6±1.8	0.02
Alzheimer's disease (%)	77.5	80.8	74.7	0.35
Education (years)	6.7±4.2	7.2±4.2	6.3±4.3	0.18
Number of children	2.0±1.5	1.8±1.4	2.1±1.5	0.11
Civil status (% Married)	54.4	56.4	52.7	0.63
Caregiver (%)Son/daughterSpouseOther family memberCare workerOther	56.237.92.40.63.0	56.437.22.6-3.8	56.038.52.21.12.2	0.86
Presence of a care worker (%)	36.7	42.3	31.9	0.16
Mean MMSE score	16.8±5.8	16.4±5.3	17.1±6.1	0.37
Mean ADL score	3.6±1.9	3.4±1.9	3.7±1.9	0.36
Mean IADL score	1.9±1.8	1.9±1.8	2.0±1.8	0.76
Use of antipsychotic drugs (%)	13.6	16.7	11.0	0.28
Use of AChE-Is (%)	47.3	52.6	42.9	0.21
Use of antidepressant drugs (%)	27.2	26.9	27.5	0.94
Use of benzodiazepine (%)	16.0	14.1	17.6	0.54
Use of memantine (%)	9.5	15.4	4.4	0.02
Aggression (%)	41.4	43.6	39.6	0.60
Psychosis (%)	28.4	25.6	30.8	0.46
Insomnia (%)	20.7	16.7	24.2	0.23

MMSE: Mini Mental State Examination.

ADL: Activity of Daily Living.

IADL: Instrumental Activity of Daily Living.

AChE-Is: Cholinesterase Inhibitors.

**Figure 3:**
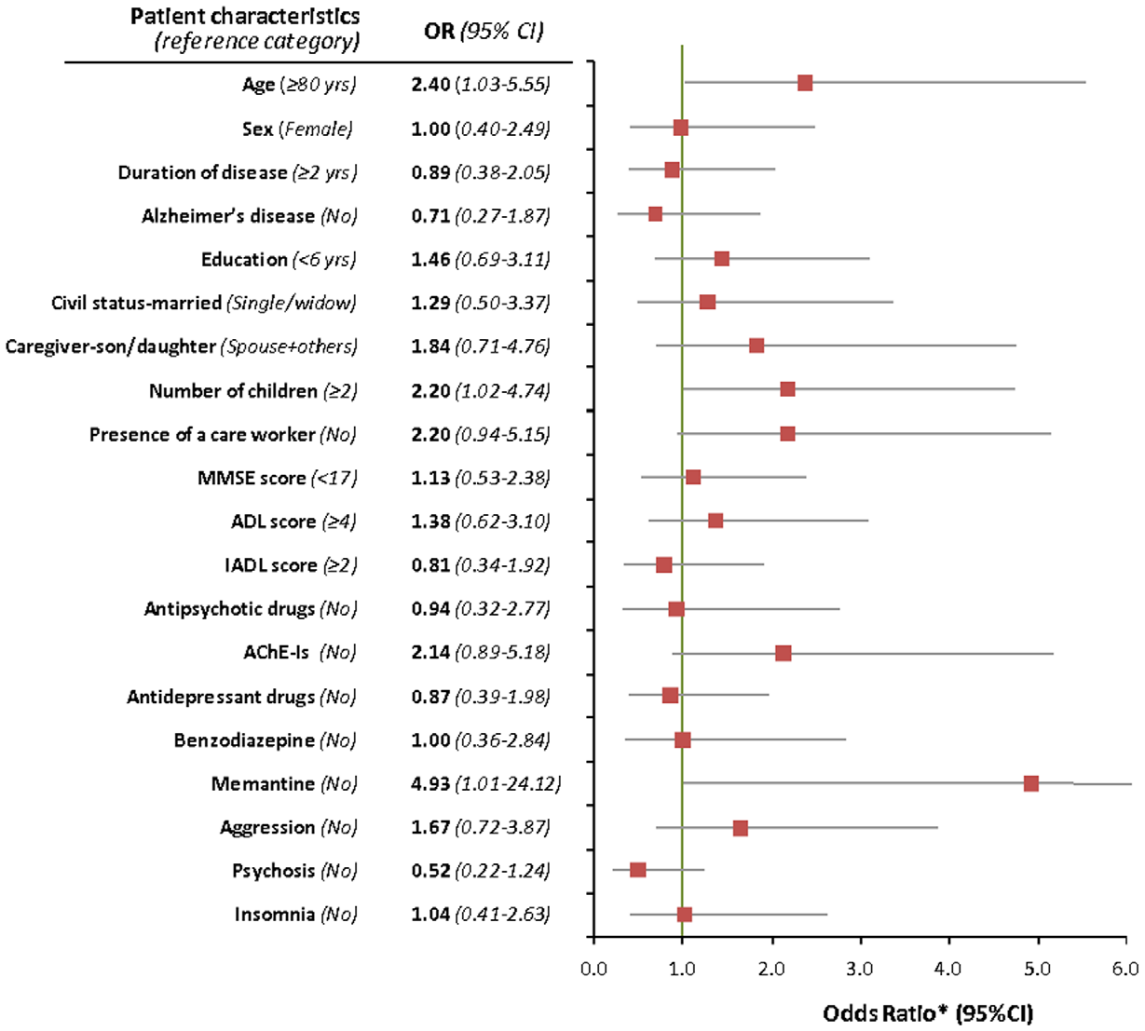
Predictive variables of appointment: results of the logistic regression analysis.

The multivariate analysis (Figure 3) shows that patients with less than two children had a higher probability to start up the procedure of appointment (OR 2.20; 95% CI 1.02–4.76) as compared to patients who had more than two children. The subjects who started up the procedure of appointment were younger (OR 2.40; 95% CI 1.03–5.53) and made more use of memantine (OR 4.93; 95% CI 1.01–24.12) than those who did not start up the procedure. Some patients made more use of AChE-Is, but the difference was not statistically significant (OR 2.14; 95% CI 0.89–5.18).

The presence of a care worker in the patient's home slightly increased the chances of appointment of the legal proxy.

We could retrieve information on the legal proxy's powers in 38 of 55 sentences (69%) of the tutelary judge. The most frequent power recognised to the legal proxy was “to keep contacts with the health authorities and to give informed consent to surgical acts, medical treatments and clinical research” (36/38; 95%), followed by “to manage the patient's property” (33/38; 87%), “to decide for the patient's living arrangements (choosing between a home and a nursing home or a residential care home)” (31/38; 82%), “to keep contacts with the tributary authority and other public bodies” (25/38; 66%).

We could retrieve information on the legal proxy's duties in 33 of 55 sentences (60%) of the tutelary judge. The most frequent duties assigned to the legal proxy were: “to give an account to the tutelary judge on the patient's property management” (28/33; 85%) and “to give an account to the tutelary judge on the patient's general conditions” (24/33; 73%).

Ninety-one patients out of 169 (53.8%) did not apply to appoint a legal proxy. Of these, 70 (76.9%) gave no specific motivations for not appointing the legal proxy. The more frequent reasons cited for not appointing a legal proxy were the following:

- disagreements among relatives about who should be the legal proxy and when to start up the procedure of appointment (9.9%);
- negative advice of GPs or other relevant figures on the opportunity to appoint a legal proxy (6.6%);
- relatives' fear of economic implications (i.e. fear that the legal proxy may take economic advantage of his or her position, or take control on the patient's fiscal situation, personal estate and legacies) (5.5%);
- relatives' fear of psychological relapses on the part of the patient (worries about upsetting him or her) (1.1%).

## Discussion

There is consensus over the fact that adults who lack capacity should be supported by proxy consent when involved in research. However, the question of who can act in this way, the extent of their authority and how they are appointed differs between countries. These inconsistencies raise questions as to which procedures best support the subject's interests; protecting them from unethical and illegal procedures, but also allowing them to potentially reap the benefits of the outcomes of the research, namely beneficial treatments.

The requirement that a legal representative support potential participants in their decision to participate to a clinical trial should ensure protection of research participants. Unfortunately, however, in Italy the requirement that the legal representative be appointed by the courts may impede a subject's participation in research for several reasons.

Within the context of a family the need to appoint one single person as the subject's legal representative may cause embarrassment and conflicts among family members.

In fact, in our study group the number of sons and daughters was a predictive variable of “non appointment” of the legal proxy. The more cited reasons for not appointing a legal proxy were the impossibility to achieve an agreement among relatives, followed by the relatives' fear that the legal proxy may take advantage of his position.

This worry seems partially justified since, even when appointment was required to allow the proxy “to keep contacts with the health authorities and to give informed consent”, in 87% of cases the tutelary judge also conferred him/her the power “to manage the patient's property”.

Further, even in a peaceful family arrangement, relatives may perceive the need to appoint a legal representative as a bureaucratic and cumbersome task. Moreover, the potential proxy may be reluctant to make difficult decisions on behalf of the potential participant, and so may postpone the decision to take on this role.

Finally, some families may perceive courts to be a stigmatizing place, thought primarily for criminal settings [33]. As such they may be reluctant to attend court as part of the process of appointing a legal proxy.

We can hypothesise that, due to the complexity and multiple implications of the procedure of appointment of the legal proxy, only some “privileged” categories of patients succeed in achieving it.

The results of our study partly support this view. We found indeed that the probability of appointing a legal proxy was associated with the younger patient's age (OR 2.40; 95% CI 1.03–5.55) and the longer duration of the patient's disease (2.3±2.2 versus 1.6±1.8 yrs; p = 0.02). This would suggest that the legal procedure is more often carried on by patients who have received an early diagnosis of dementia. Precocity of the diagnosis may be indicative of the patients' and relatives' stronger attention to the symptoms of dementia as well as their prompter access to the healthcare services. Prompt access to the healthcare services may be related to the patients' and relatives' more confident access to other public services, including the law courts. This is compatible with the slightly higher educational level of the patients who started up the procedure of appointment of a legal proxy.

Our data also suggest that the procedure of appointment was carried out by the patients who had a more stable and lasting relationship with our clinical centre.

This view seems supported by the statistical association among the appointment of a legal proxy and the patient's use of memantine (OR 4.93; 95% CI 1.01–24.12) and a quasi-significant association with the patient's use of AChE-Is (OR 2.14; 95% CI 0.89–5.18). Indeed, when the AdCare study was started, in the Lombardy region memantine was gratuitously distributed from clinical centres participating to a project [34] coordinated by the Centre for Research and Treatment on Cognitive Dysfunctions, “L. Sacco” Hospital [35]. Since July 2005 the patients who were taking memantine had been entering three-monthly to the centre to verify the efficacy and tolerability of the treatment and to receive memantine for the following three months. The patients who were taking cholinesterase inhibitors had been entering to the centre every six-month as provided by the Italian regulation on the reimbursement of these treatments. Hence, we can presume that the patients and relatives who had more promptly appointed a legal proxy for the patient were the patients who had a strong and fiduciary relation with the neurologist, as they were stably related to the centre by the system for reimbursement of pharmacological treatments. As compared to other patients, these patients accede to the Centre with:

- more frequent scheduled visits;
- direct reservation with the centre (instead of reservation by the regional call-centre)
- availability of a mobile phone number to call at any time to notify adverse drug reactions.

This “preferential” treatment may have contributed to reinforce the trusting relationship among patients, their caregivers and the centre thus determining a more favourable attitude towards the suggestions of the centre's staff, including the suggestion to provide legal agency to the patient.

It is to note that the appointment of a legal proxy would have had no advantage nor any disadvantage for these patients as regarding the possibility to receive a beneficial treatment.

As regarding reasons for not appointing a legal proxy, besides relatives' reluctance to start up the procedure, we identified another obstacle, which is the time required to complete all the procedure once started. In fact, our data show that the median time required to carry out all the proceeding is on average twofold (median time 121 days) than that previewed by the law (60 days). Hence, the times required by the courts to appoint a legal representative may not be synchronised with the times required for an individual's participation in research.

For all these reasons the system which is actually in place in Italy seems far from effective in balancing the needs of protection of subjects and the need of rapid times for subject's enrolment in research.

Indeed, the difficulties in recruiting patients in AdCare, mostly due to complexities in the informed consent procedure, determined the anticipated interruption of the trial.

The data that we provide here refer to one of the most active centres participating to the AdCare study, and one related to a well functioning court. Following the same procedure for informed consent, many other centres participating to the AdCare study didn't succeed in recruiting not even one patient.

If we should think how to change the system in place we could take example from other EU countries systems. For example, the pragmatic approach adopted in the Belgian, Dutch, French or Spanish legislation, providing proxy consent from a hierarchy of family members when a legal representative is not available, seem more encouraging towards clinical research than appointing a representative through the courts, being less time requiring. Results of our study show indeed that, conformingly to the law, the legal proxies appointed by the courts are patient's relatives in 100% of the cases. Thus, from a pragmatic point of view, the adoption of a juridical proceeding to confirm what naturally happens among the patient's families may seem redundant.

However, relatives' consent may be less protective towards subjects in that less control would be given to the procedure of their appointment. Nevertheless, we know of no data supporting the idea that more control, for example through the involvement of courts, allows better protection towards potential research participants.

Another possibility would be to empower the offices of the tutelary judges in courts to fasten the times required to appoint the legal proxies. Empowering courts however would require to invest more financial and human resources in the Italian court system. Moreover, this would not solve the other obstacle that we observed in our study that is relatives' reluctance to appoint a legal proxy.

Another way to ensure protection to research participants would be to empower the role of GPs as some legislation provides. The role of ethics committees may also be strengthened. In the Netherlands a central committee for medical research reviews the protocols for certain types of research (without direct benefit) involving people who are unable to give informed consent [17]. Also in the UK, the ethical review of research protocols involving incapacitated adults is made by specialised ethics committees [36].

Doctors and ethics committees are likely to have the expertise to judge whether or not a proposed trial is in the best interest of participant. However, doctors and ethics committees might have potential conflict of interests with their colleagues or with the drug industry [37], [38] and select the legal representative who is more favourable to patient's involvement in research.

On the contrary, courts may have a more neutral and impartial approach towards appointing the proxy which better represents the patient's interests, but the involvement of courts, as we have demonstrated, slows down the process of appointment.

Additional protections may be important to safeguard participants' welfare. Nevertheless, the role played by a potential participant's autonomy and the principle of informed consent are irreplaceable in the ethics of clinical research.

More studies should be devoted to the practice of proxy consent and to questions regarding the extent to which formal designation of a proxy does guarantee a more accurate representation of a participant's wishes. Divergent views emerge in this respect. Some studies show that elder persons and persons at risk of developing Alzheimer disease are generally supportive of surrogate consent for participation in research [39], [40]. Other studies show that often there is only fair agreement between what a proxy thinks a patient would decide and what the patient actually decides in his or her care [41], [42].

Ideally a proxy should have a clear understanding of a potential participant's wishes with respect to their involvement in research. To enhance understanding it will be important that doctors, patients and their caregivers discuss the possible evolution of the disease and any potential opportunities for participation in a clinical trial at an early stage of the disease's development [43]. This will allow the proxy to be more prepared should such an opportunity arise in the future. This 'learning and preparation' can be achieved through the relationships among potential participants, proxies, doctors and other healthcare providers but also in the wider arena of public debate.
